# Supplementary material for: Ion identity molecular networking for mass spectrometry-based metabolomics in the GNPS environment
Source: Nat Commun. 2021 Jun 22;12:3832. doi: 10.1038/s41467-021-23953-9 (PMC8219731; doi:10.1038/s41467-021-23953-9)
Supplement: Supplementary file 1 — Supplementary Information [file 41467_2021_23953_MOESM1_ESM.pdf]

Supplementary Information

Ion Identity Molecular Networking for mass spectrometry-based metabolomics in the GNPS environment

Robin Schmid[1,2#], Daniel Petras[2,3#], Louis-Félix Nothias[2#], Mingxun Wang[2], Allegra T. Aron[2], Annika Jagels[4], Hiroshi Tsugawa[5,6], Johannes Rainer[7], Mar Garcia-Aloy[7], Kai Dührkop[8], Ansgar Korf[1], Tomáš Pluskal[9], Zdeněk Kameník[10], Alan K. Jarmusch[2], Andrés Mauricio Caraballo-Rodríguez[2], Kelly C. Weldon[2], Melissa Nothias-Esposito[2], Alexander A. Aksenov[2,11], Anelize Bauermeister[2,12], Andrea Albarracin Orio[13], Carlismari O. Grundmann[2,14], Fernando Vargas[2], Irina Koester[3], Julia M. Gauglitz[2], Emily C. Gentry[2], Yannick Hövelmann[4], Svetlana A. Kalinina[4], Matthew A. Pendergraft[3], Morgan Panitchpakdi[2], Richard Tehan[15], Audrey Le Gouellec[16], Gajender Aleti[17] , Helena Mannochio Russo[2,18], Birgit Arndt[4], Florian Hübner[4], Heiko Hayen[1], Hui Zhi[19], Manuela Raffatellu[19,20], Kimberly A. Prather[3], Lihini I. Aluwihare[3], Sebastian Böcker[8], Kerry L. McPhail[15], Hans-Ulrich Humpf[4], Uwe Karst[1], Pieter C. Dorrestein[2,11]*

1. Institute of Inorganic and Analytical Chemistry, University of Münster, Münster, Germany
2. Skaggs School of Pharmacy and Pharmaceutical Sciences, University of California San Diego, La Jolla, San Diego, CA, USA
3. Scripps Institution of Oceanography, University of California San Diego, La Jolla, CA, US
4. Institute of Food Chemistry, University of Münster, Münster, Germany
5. RIKEN Center for Sustainable Resource Science, Yokohama, Kanagawa, Japan
6. RIKEN Center for Integrative Medical Sciences, Yokohama, Kanagawa, Japan
7. Institute for Biomedicine, Eurac Research, Affiliated Institute of the University of Lübeck, Bolzano, Italy
8. Chair for Bioinformatics, Friedrich-Schiller-University, Jena, Germany
9. Institute of Organic Chemistry and Biochemistry, Czech Academy of Sciences, Prague, Czech Republic
10. Institute of Microbiology, Czech Academy of Sciences, Prague, Czech Republic
11. Collaborative Mass Spectrometry Innovation Center, University of California San Diego, La Jolla, San Diego, CA, USA
12. Institute of Biomedical Sciences, Universidade de São Paulo, São Paulo, SP, Brazil
13. IRNASUS, Universidad Católica de Córdoba, CONICET, Facultad de Ciencias Agropecuarias. Córdoba, Argentina
14. School of Pharmaceutical Sciences of Ribeirão Preto, Universidade de São Paulo, Ribeirão Preto, SP, Brazil
15. Department of Pharmaceutical Sciences, College of Pharmacy, Oregon State University, Corvallis, OR, USA
16. Univ. Grenoble Alpes, CNRS, Grenoble INP, CHU Grenoble Alpes, TIMC-IMAG, Grenoble, France
17. Department of Psychiatry, University of California San Diego, San Diego, CA, USA

18. NuBBE, Institute of Chemistry, São Paulo State University (UNESP), Araraquara, SP, Brazil
19. Division of Host-Microbe Systems & Therapeutics, Department of Pediatrics, University of California San Diego, La Jolla, CA, USA
20. Chiba University-UC San Diego Center for Mucosal Immunology, Allergy and Vaccines (CU-UCSD cMAV), La Jolla, CA, USA

*Correspondence should be addressed to pdorrestein@ucsd.edu
# These authors contributed equally

Content:

Page 3-4: Supplemental Notes
Page 5-9: Supplemental Figures
Page 10-19: Supplemental Tables
Page 20-21: Supplemental References

Supplementary Note 1: Metal-binding compounds and ionophores

Ion identity molecular networking can be used in combination with native ESI-based metabolomics[12] to find biologically-relevant metal-binding compounds or to elucidate metal-binding preferences of known or novel metal-binding molecules (Zhi, H. et al., submitted). One recent example in which IIMN was instrumental in understanding metal-binding and selectivity is yersiniabactin. We identified yersiniabactin as a novel zincophore produced by E. coli Nissle by performing post-liquid chromatography (LC) pH adjustment (to pH 6.8) and infusion of zinc acetate solution, followed by mass spectrometry and ion identity molecular networking. With this strategy, mass spectrometry features with correlated peak shapes and retention times, in addition to an *m/*z difference resulting from zinc-binding (+Zn^{2+} -H^{+}) were found. These results are summarized in Supplementary Figure 3. While this example highlights the discovery of a zinc-binding molecule explicitly, IIMN has been used in conjunction with the infusion of other metals, including iron, copper, and cobalt, to find siderophores and other ionophores. Molecular formulas were predicted with SIRIUS 4.0 after exporting MS^1 isotope pattern and MS^2 spectra as a data processing step in MZmine. The IIMN job can be accessed on GNPS (https://gnps.ucsd.edu/ProteoSAFe/index.jsp?task=525fd9b6a9f24455a589f2371b1d9540).

Supplementary Note 2: Compound structure information

The ion identity molecular networking results for the *Stachybotrys chartarum* dataset (MSV000084134) prove that the ion identity annotations can yield structure relevant information. Putative molecular formula modifications (+O and +H_2O) between chemical compounds can be verified by the maximum number of water losses that were annotated by IIN. The difference in the number of oxygens in the molecular formulas of phenylspirodrimane derivatives is reflected in additional losses of H_2O within the corresponding IINs. The results are depicted in Supplementary Figure 5 as combined IIMN networks (a) and with collapsed ion identity networks (b). The IIMN job can be accessed on GNPS (rerun of the original job after additional spectral library entries were added to the GNPS spectral libraries: https://gnps.ucsd.edu/ProteoSAFe/status.jsp?task=3bd4def5e0e348c9b113f4a072f03ea9).

Supplementary Note 3: Implementation of orthogonal supplementary edges

Ion identity molecular networking was the initial driver to implement the option of supplementary edges into the FBMN workflow on GNPS. However, based on the generic format, any tool can create and export new relationships between features to link the corresponding nodes in feature-based molecular networks. As an example, we have implemented a new MZmine module to annotate neutral mass differences between ion identity networks as putative chemical modifications, in the format of supplementary edges. These edges connect two IIN if the neutral mass difference matches a user-defined modifications list.

The IIMN MZmine workflow was applied to a dataset of 88 bile acid extracts from feces and gall bladder of various animals (MSV000084170). IIN modification edges were based on the mass differences of +methyl (Me, CH_2), +O, and +H_2O. To exemplify the results, Supplementary Figure 6 shows a network cluster of glycocholic acid analogs. Library matching annotated most of the ion identity networks as glycine conjugated bile acids; Two IINs as glycocholic acid (+isomers) and two IINs as glycodeoxycholic acid (+isomers) with a mass accuracy of <2 ppm. The additional modification edges connect these structurally related compounds and increase the network density. Moreover, they help to infer putative molecular formulas and modified structures from an FBMN. In a second analysis of the same dataset, IINs were connected based on mass differences of the modification by taurine, glycine, and alanine conjugation. This resulted in additional links between conjugated and free bile acid forms of cholic acid and deoxycholic acid. The IIMN jobs can be accessed on GNPS.

IIMN
https://gnps.ucsd.edu/ProteoSAFe/status.jsp?task=0a3f4399e5344188805e5856b756d918
IIMN: Methyl (Me, CH_2), O, and H_2O modification edges
https://gnps.ucsd.edu/ProteoSAFe/status.jsp?task=465d7285380942a0828e462d1db027c2
IIMN: Taurine, glycine, and alanine modification edges
https://gnps.ucsd.edu/ProteoSAFe/status.jsp?task=69b40f808b2047d89fccf3d07e79fc59

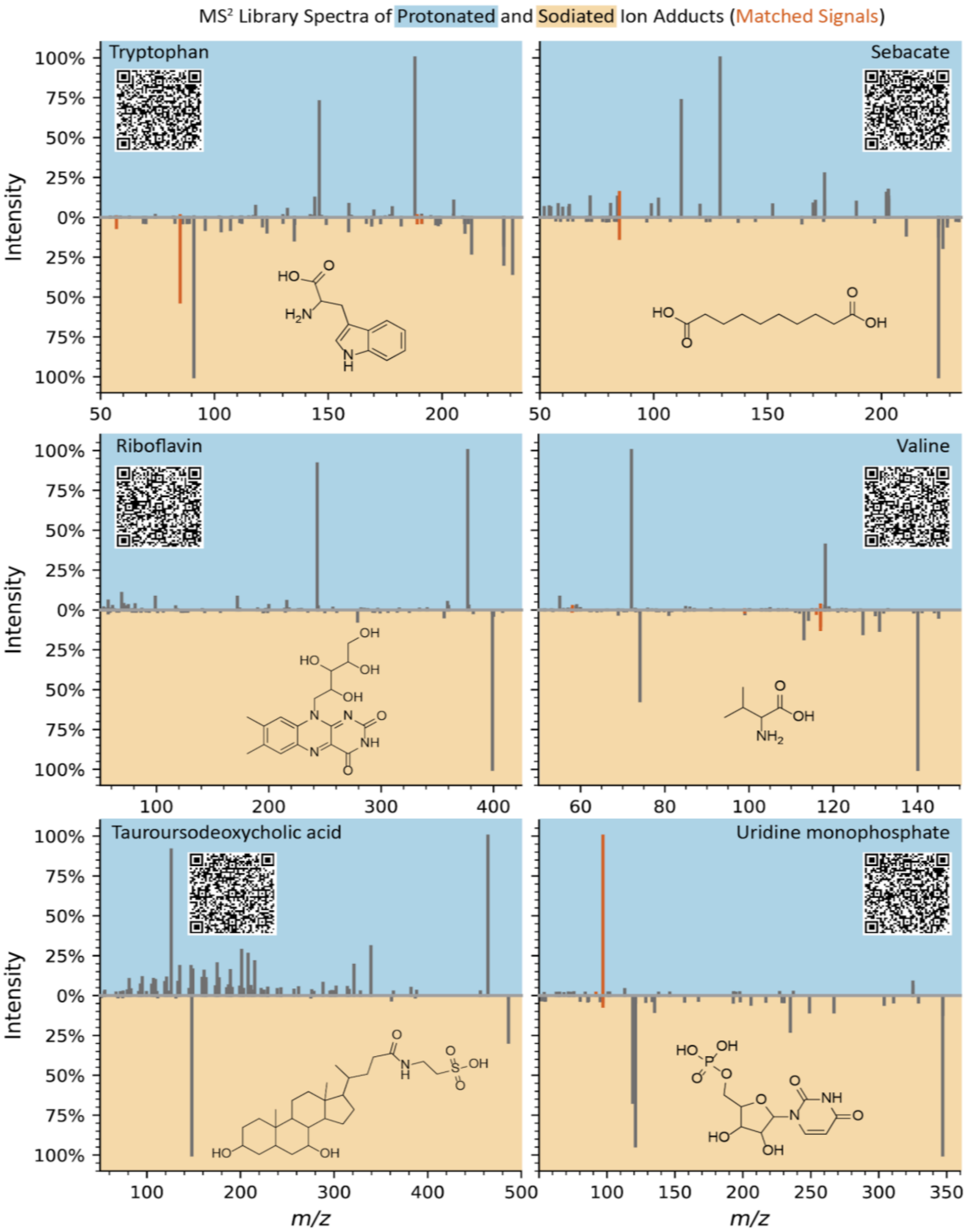

Supplementary Figure 1: Comparison of MS^2 library spectra for sodiated and protonated ion species of six compounds. The spectral mirror charts show MS^2 library spectra of the same compound as $[M+H]^+$ (top plot) and $[M+Na]^+$ (bottom plot). Due to high in-source fragmentation and low $[M+H]^+$ abundance, tauroursodeoxycholic acid compares the $[M-H_2O+H]^+$ and $[M+Na]^+$ MS^2 library spectra. Matched signals in mirror charts are highlighted (dark orange). These charts exemplify a commonly seen difference in fragmentation behavior for sodiated and protonated ion adducts. All library spectra are publicly available in the GNPS spectral libraries. The charts were created with the Metabolomics Spectrum Resolver (https://metabolomics-usi.ucsd.edu/) and scannable QR-codes link out to the individual annotated charts and library spectra with their metadata.

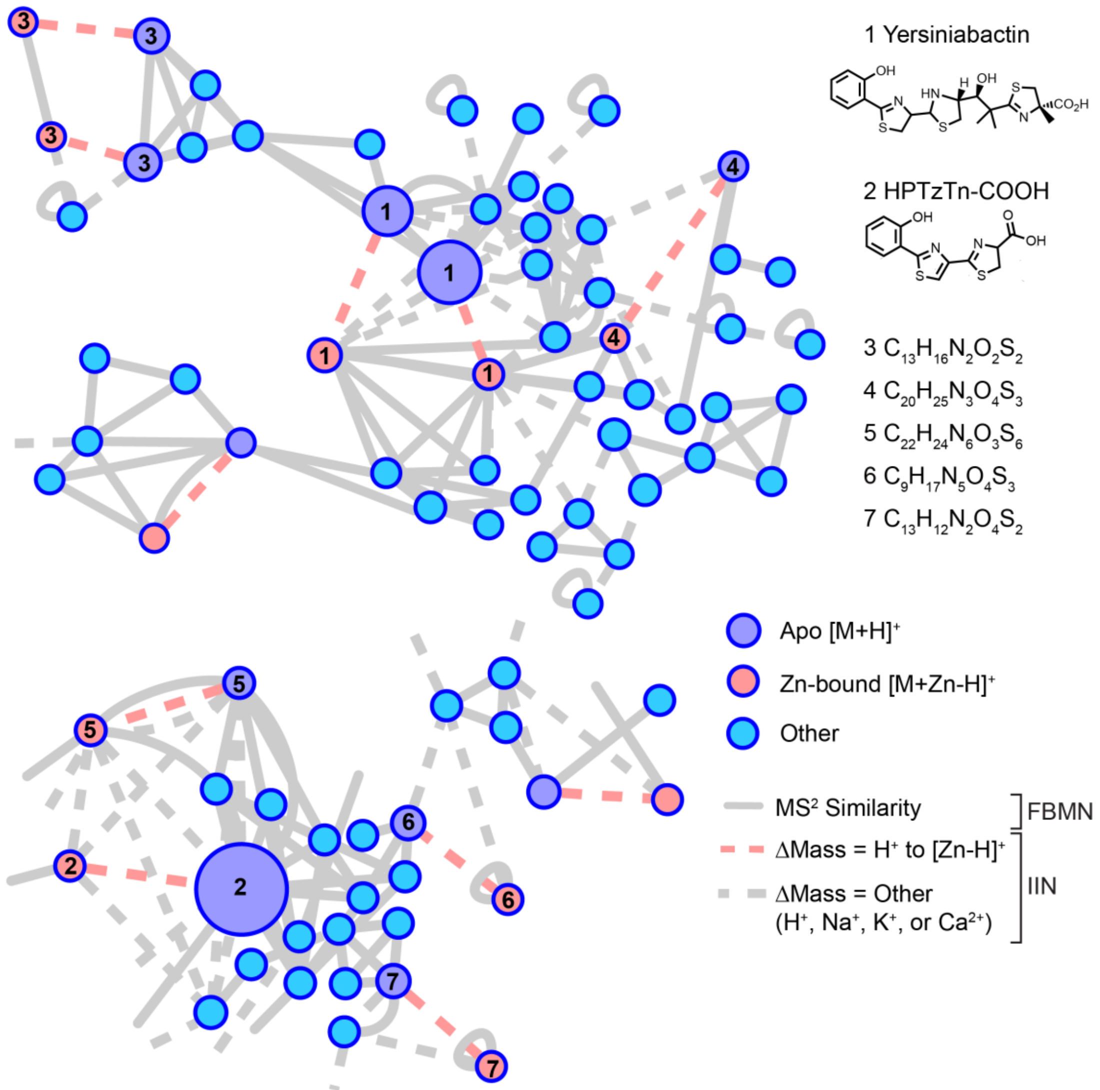

Supplementary Figure 2: Use case for the discovery of metal-binding compounds and ionophores. IIMN in conjunction with native spray metal metabolomics[12] facilitated the discovery of yersiniabactin as a zincophore produced by *E. coli* Nissle (Zhi, H. et al., *submitted*). Zinc-binding molecules, such as yersiniabactin and other potential derivatives or truncations, are shown in salmon, while the corresponding protonated form of these molecules is shown in purple; other nodes are colored light blue. Structures and molecular formulas (generated using SIRIUS 4.0) for compounds 1-7 are provided.

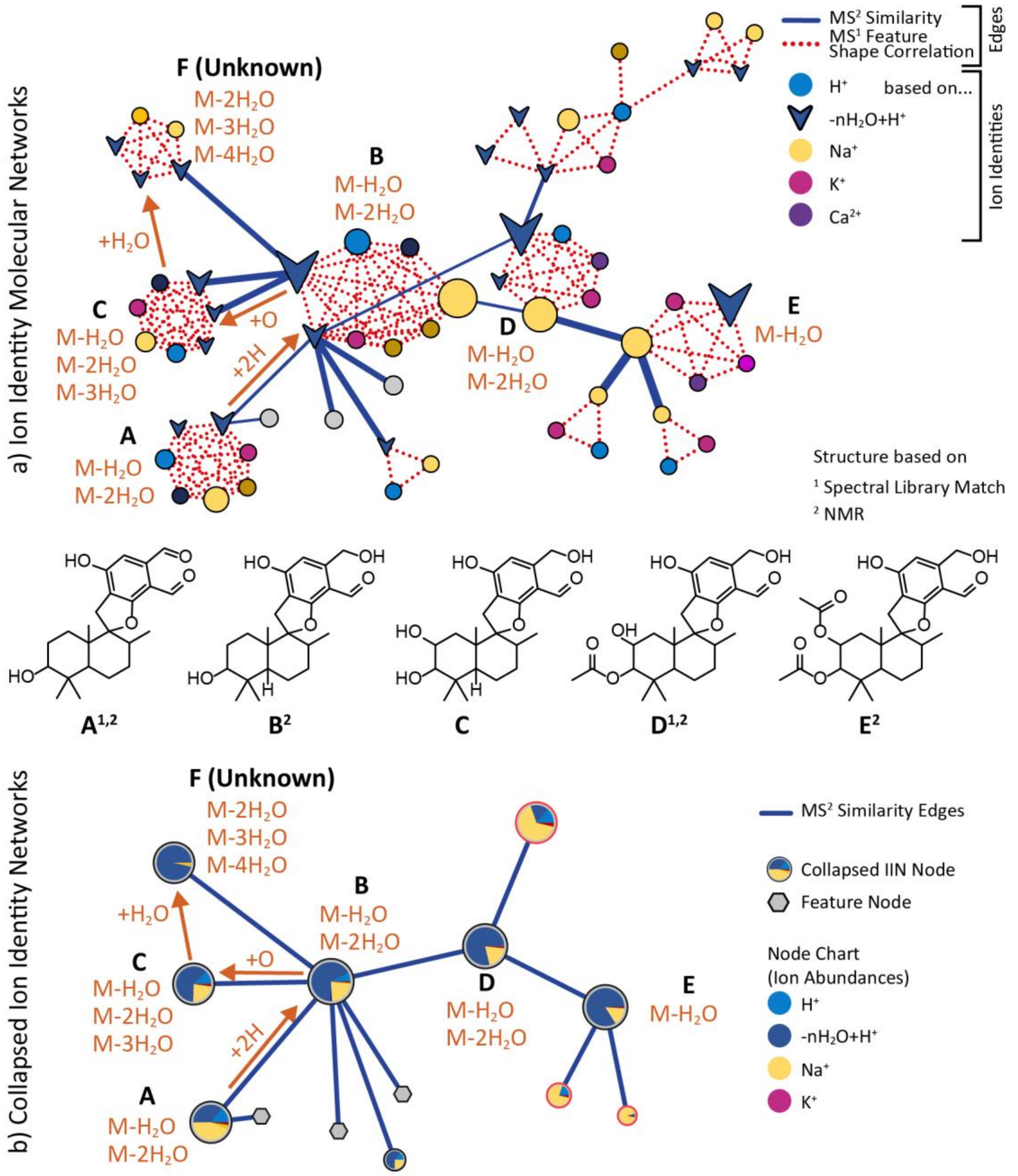

Supplementary Figure 3: Ion identity molecular networking results for *Stachybotrys chartarum* liquid culture extracts (MSV000084134). The presented two visualization options compare the combined ion identity molecular network (a), with all ion identities of the same compound sorted in circles, and a collapsed IIN network version (b), with pie-charts representing the relative ion abundances of the corresponding compounds. Compounds A and D were annotated by spectral library match and compounds A, B, D, and E were verified by nuclear magnetic resonance (NMR) spectroscopy[27]. Modifications between compounds are based on the compound structures or the differences of the average neutral masses for each IIN. The modifications of +O and +H_2O from B to C to F can also be deduced from the ion identity annotations, as the maximum in-source water losses for each compound confirm the addition of oxygens; Compounds A, B, and D (-2H_2O maximum water losses), compound C (-3H_2O), and compound F (-4H_2O). Therefore, this example proves that IIN can yield structure relevant information which facilitates structure annotation and propagation.

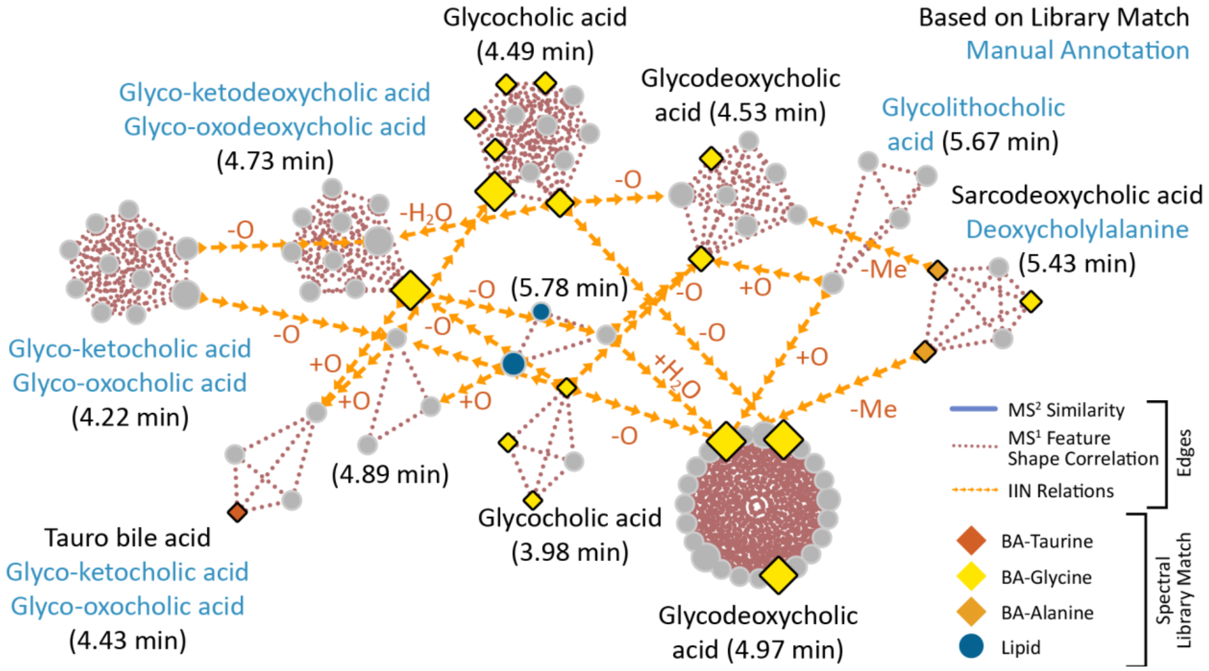

Supplementary Figure 4: Use case for supplementary edges to spot compound modifications. Ion identity networks were linked by additional edges based on neutral mass differences matching those of methyl (Me, CH_2), O, or H_2O. MS^2 spectral similarity edges are hidden to reduce visualization complexity. Spectral library matching annotated two IINs as glycocholic acid (+isomers) and two IINs as glycodeoxycholic acid (+isomers). The new additional modification edges helped to infer putative annotations for six IINs, moreover, in the resulting IIMN, related compounds clustered closer and with more connections.

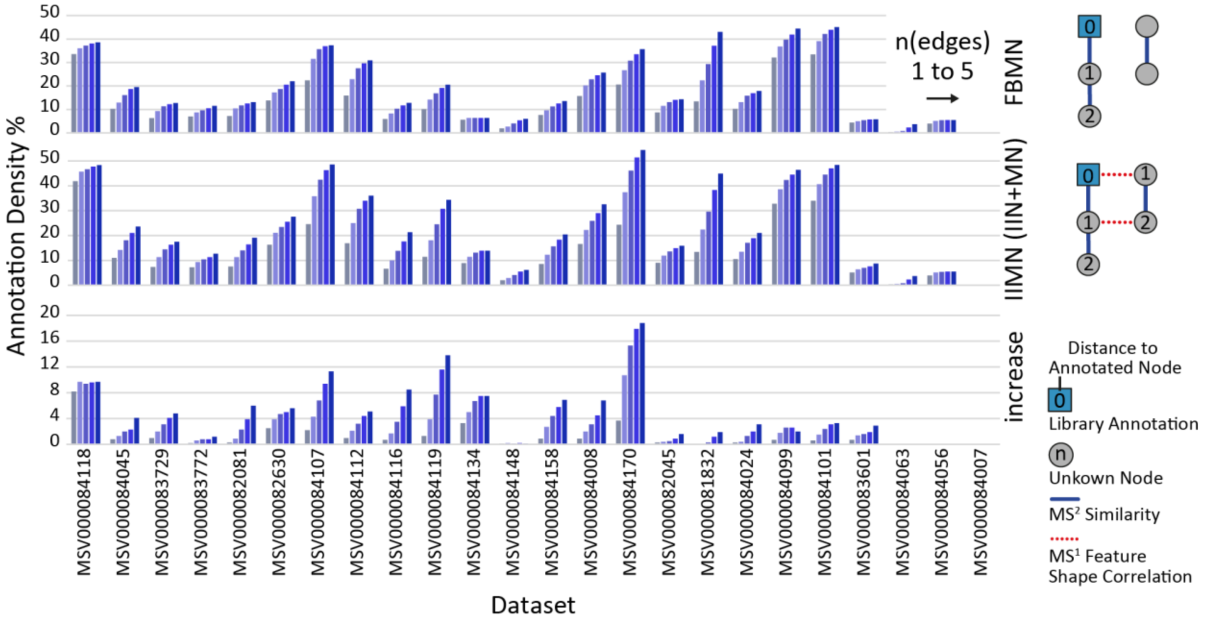

Supplementary Figure 5: Compound annotation densities for maximum distances over 1 to 5 edges to an annotated node. From top to bottom, nodes that are connected to at least one annotated compound by n FBMN edges (top), by IIMN (IIN+FBMN) edges (middle), and the differential increase of annotation density by adding IIN to FBMN (bottom). The increase over one edge corresponds to unknown features (nodes) which are directly connected by an IIN edge to an annotated compound (maximum increase +8%). Source data are provided as a Source Data file.

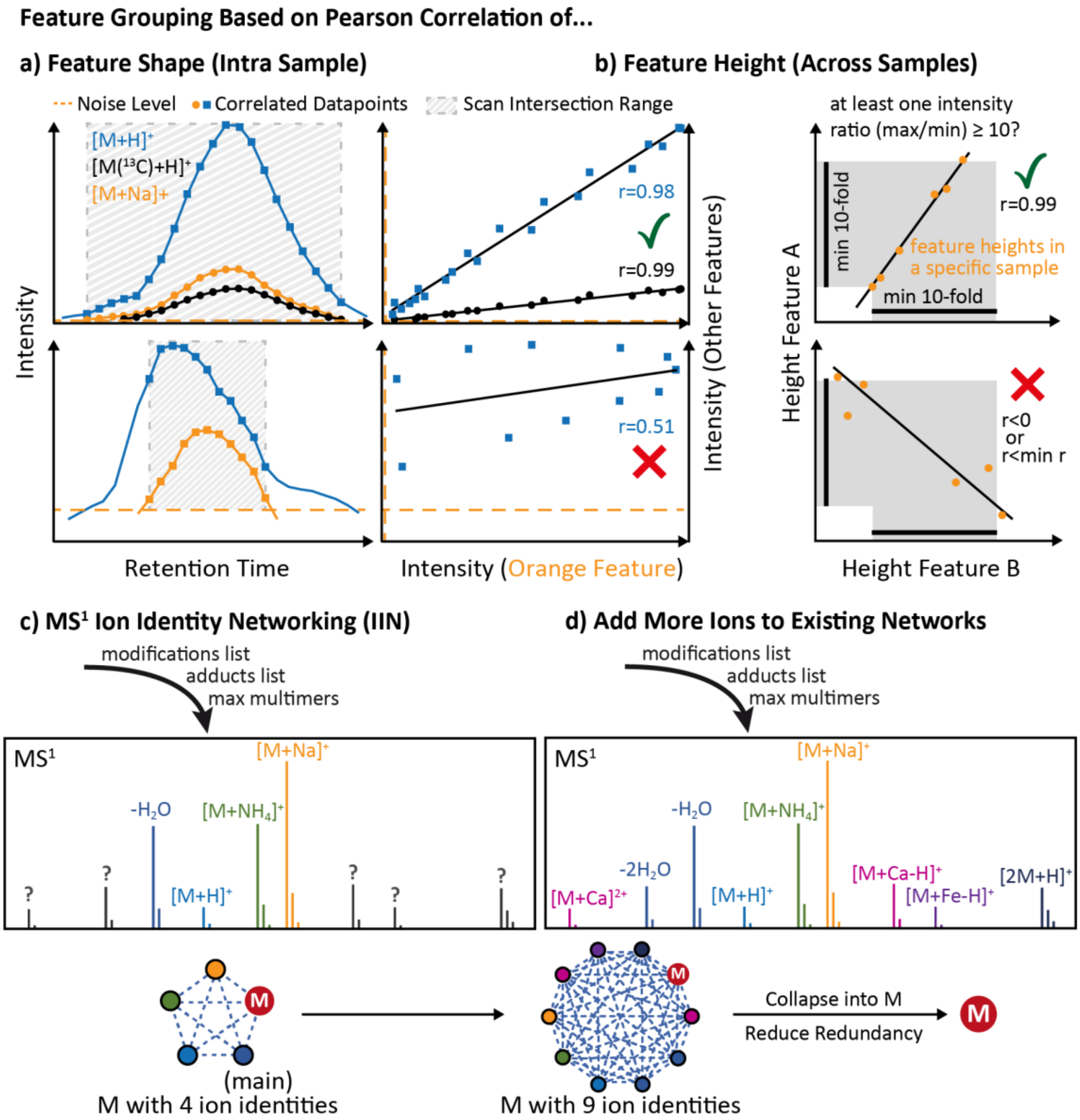

Supplementary Figure 6: Schematic workflow for feature grouping (metaCorrelate) and ion identity networking within MZmine. After preselecting features that are in a retention time window and have a minimum overlapping retention time percentage (measured on the smaller feature for each tested pair), features are grouped if **a)** their shapes (intensity profiles) and **b)** their heights across all samples resolve to Pearson correlation coefficients (r) of user-defined thresholds. Optionally, both correlation filters can be omitted or switched to a cosine score similarity threshold. By turning off both filters, the grouping is solely based on the retention time window and overlap prefilters. Ion identity networking uses an in-source modifications list, an adducts list, and a “maximum multimers number” parameter to create a library of MS^1 ion identities. To create annotation networks, all pairs of ion identities are applied to all pairs of grouped features to calculate the neutral mass of the corresponding feature (*m/z*) with an ion identity (mass difference, charge (*z*), and multimer number). After **c)**, the initial creation of ion identity networks with a user-defined library of main ion identities, more uncommon ion identities can **d)** be added iteratively to existing networks.

Supplementary Table 1: LC-MS^2 datasets used for the evaluation of ion identity molecular networking. All datasets are publicly available through their corresponding ID in the MassIVE repository (massive.ucsd.edu).

#	Dataset Name	Sample Origin	Dataset Description	Organism Name	MassIVE ID
1	B. Sub knockout	*B. subtilis*	Extracts from B. sub wildtype and srf-KO cell culture	*Bacillus subtilis*	MSV000082081
2	Coral Reef Sea Water	Seawater	Spatial survey of Coral Reef in Hawaii, filtered and unfiltered samples	Seawater	MSV000084116
3	Drug Metabolism Tsunoda	Human	Human blood (plasma) prepared using Phree kit, individuals given 4 different drugs (omeprazole, dextromethorphan, caffeine, and midazolam)	*Homo sapiens*	MSV000084008
4	SD Cheetahs	Cheetah	Fecal samples from cheetahs in human care	*Acinonyx jubatus*	MSV000084099
5	Nascent Sea Spray Aerosol	Seawater	isolated sea spray aerosol	Seawater	MSV000084119
6	Fungus-Growing T. septentrionalis Gardens	Ants fungus garden extracts	DCM:MeOH 2:1 extracts from Trachymyrmex septentrionalis fungus gardens.	*Trachymyrmex septentrionalis*	MSV000084024
7	HMP cultures full set	Various bacterial cultures	Extracts of HMP project isolates that were grown successfully in three different media	433 various bacteria	MSV000082045
8	Foodomics subset	Diverse foods	Subset of food samples from Global FoodOmics project	Various foods	MSV000084101
9	Stromatolites tissue	Intertidal - freshwater/marine stromatolites	Extracts from South African stromatolite cores, and San Diego	Cyanobacteria/Heterotrophic bacteria	MSV000083729
10	Stromatolites DOM	Intertidal - freshwater/marine DOM	DOM/TOM analysis of water from SA stromatolite barrage pool	Cyanobacteria/Heterotrophic bacteria	MSV000083772
11	Bacteria from Rocas Atoll	Sediments from Rocas Atoll	Extracts produced by marine bacteria in liquid cultures	Bacteria	MSV000083601
12	B. subtilis extracted cell-free supernants	*B. subtilis*	Methanol extracts from cell-free supernatants of B. subtilis wild strains	*Bacillus subtilis*	MSV000084045
13	SEED Grant - Sejal - Urine	Human	Urine samples extracted in 80% MeOH of Disease-associated microbial species, for disease diagnosis and prevention of Type I diabetes	*Homo sapiens*	MSV000084112

Supplementary Table 1: continued

#	Dataset Name	Sample Origin	Dataset Description	Organism Name	MassIVE ID
14	ONR Primary Wright - Human-Plasma	Human	Dataset investigating the impact of sleep and circadian disruption on human plasma	*Homo sapiens*	MSV000082630
15	Basidiobolus	Fungi, Zoopagomycota	MeOH extract/RP-SPE fractions of B. meristosporus, grown in 7 growth conditions in triplicate. EtOAc extract/RP-SPE fractions of culture broths. Study aims to discover the product of bacterial-like NRPSs.	*Basidiobolus meristosporus*	MSV000084007
16	Pseudonocardia Extracts Grouping - Illumina	*Pseudonocardia*	EtOAc extracts of 7 Pseudonocardia strains to discover if there some kind of grouping according to metabolomic content	*Pseudonocardia*	MSV000084056
17	R_HAN_01.05	*Mus musculus*	Plasma serum from mice fed with prebiotics or antibiotics for 3 weeks	*Mus musculus*	MSV000084107
18	Tunicates - Euherdmania X Eudistoma vannamei	Marine organisms	Extraction with MeOH of macerated organism	*Euherdmania* and *Eudistoma vannamei*	MSV000084063
19	Saliva samples from caries and healthy children	Human	Saliva was lyophilized and non-polar molecules were extracted in ethyl acetate	Oral microbiome	MSV000081832
20	Stachybotrys-WT-OE	Filamentous fungi	Extracts from mycelium and filtates	*Stachybotrys chartarum*	MSV000084134
21	Urine biomarker discovery	Human	Diluted urine	*Homo sapiens*	MSV000084148
22	SeaScape2019 Bloom1+2	Seawater	Non-targeted metabolomics (DOM, 02 um surpore) from bulk water samples (1L) from SeaScape2019 Bloom1+2 from PPL(200mg) solid phase extraction. [doi:10.25345/C5HH23]	Seawater	MSV000084158
23	Animal bile acids	Animals (Lee Hagey collection)	Methanolic extracts of gall bladder or feces	Various *vertebrata*	MSV000084170
24	Adduct induction	Natural Products	Post-column salt addition	Standards	MSV000084118

Supplementary Table 2: Summary of statistical results on all 24 datasets. Refer to the provided Microsoft Excel workbook (SI_IIMN_dataset_statistics.xlsx) for a full dataset resolved statistical summary and in-depth results for each dataset. Source data are provided as a Source Data file.

Statistical measure	Mean %	Median %	Min %	Max %
Library matches	6	4	0	23
Ion identities	14	14	2	43
Ion identities with MS^2	12	11	2	35
Library matches with ion identity	16	16	0	75
Singletons (only FBMN edges)	43	39	23	91
Singletons<br>(all edges)	38	34	22	80
Reduced singletons	5	3	0	30
Nodes reduced by IIN	9	8	1	23

Supplementary Table 3: LC-MS^2 spectral library summary. For the statistical analysis of the ion identity coverage in available online resources, different spectral libraries were downloaded from MassBank of North America (MoNA) and GNPS and compared to the IIMN-based library, from the described workflow. MSMS-Chooser is a new workflow in the GNPS environment that focuses on the creation of spectral libraries with broader coverage of different ion identities. The currently available libraries based on MSMS-Chooser mainly contain spectral MS^2 entries for bile acids. All resources were downloaded on Dec, 17th 2019.

Library Group	Library Name	Link
MoNA[25]	LC-MS^2 (positive)	https://mona.fiehnlab.ucdavis.edu/downloads
GNPS[2]	• GNPS Library<br>• FDA Library Pt 1<br>• FDA Library Pt 2<br>• PhytoChemical Library<br>• NIH Clinical Collection 1<br>• NIH Clinical Collection 2<br>• NIH Natural Products Library Round 1 (NIH)<br>• NIH Natural Products Library Round 2 (NIH NPAC ACONN)<br>• Pharmacologically Active Compounds in the NIH Small Molecule Repository<br>• Faulkner Legacy Library provided by Sirenas MD<br>• EMBL Metabolomics Core Facility (EMBL MCF)<br>• Pesticides<br>• Medicines for Malaria Venture Pathogen Box<br>• LDB Lichen Database<br>• GNPS Collections Miscellaneous<br>• GNPS Collections Bile Acid Library 2019<br>• MIADB Spectral Library	https://gnps.ucsd.edu/ProteoSAFe/libraries.jsp
MSMS-Chooser-based (GNPS)[26]	• GNPS-MSMLS<br>• GNPS Collections Bile Acid Library 2019	https://gnps.ucsd.edu/ProteoSAFe/gnpslibrary.jsp?library=GNPS-MSMLS<br>https://gnps.ucsd.edu/ProteoSAFe/gnpslibrary.jsp?library=BILELIB19
IIMN-based	• Ion identity molecular networking-based library extracted from 24 publicly available datasets<br>• Ion identity molecular networking-based library generated from the NIH Natural Products Library (NIH NPAC ACONN). All spectral entries were added to the existing manually created GNPS library.	https://gnps.ucsd.edu/ProteoSAFe/gnpslibrary.jsp?library=GNPS-IIMN-PROPOGATED<br><br>https://gnps.ucsd.edu/ProteoSAFe/gnpslibrary.jsp?library=GNPS-NIH-NATURALPRODUCTSLIBRARY_ROUND2_POSITIVE

Supplementary Table 4: MZmine parameters for LC-MS^2 dataset processing.

ID	Samples	MS Instrument	MS Resolution	Gradient [min]	Duty Cycle Time	MS^1 threshold	MS^2 Threshold	MS Tolerance	RT Tolerance [min]	Chromatogram Deconvolution Algorithm	MS^2 Paring *m/z* Tolerance	MS^2 Paring RT Tolerance [min]	Feature Aligment (m/z Tolerance, RT Tolerance, m/z : RT Weights)	Duplicate Row Filter	Min. Peaks in a Row	Min. Isotope Pattern Peaks	MS^2 Filtered	Gap Filled
MSV000082081	7	Q-Exactive	17000	5	<1sec	1E5	1E3	10 ppm	0.02	Baseline cut-off (Min peak height: 3E5, Peak dur 0.01 - 3 min, Baseline level 1E5)	0.01	0.1	10 ppm, 0.2 min, 75:25	no	2	2	MS2 filter	yes
MSV000084116	64	Q-Exactive	17000	15	<1sec	1E5	1E3	10 ppm	0.02	Baseline cut-off (Min peak height: 3E5, Peak dur 0.01 - 3 min, Baseline level 1E5)	0.02	0.1	10 ppm, 0.15 min, 75:25	no	2	2	no	yes
MSV000084008	439	QToF/ Maxis Impact HD		7.5		1E3	1E2	0.01 m/z or 20 ppm	0.05	Local minimum search (5.0%, 0.05 min, 5.0%, 3000, 2, 0.05 - 2.00 min)	0.025	0.01	0.01 m/z or 20.0 ppm, 0.1 min, 50:25	yes	2	2	only with MS2 or annotation	yes
MSV000084099	556	QToF/ Maxis Impact HD		12.5		2E3	9E1	0.001 m/z or 20 ppm	0.03	Local minimum search (96%, 0.03 min, 5.0%, 2000, 1, 0.0 - 2.00 min)	0.02	0.15	0.0015 m/z or 15 ppm, 0.2 min, 2:1	yes	2	2	no	no
MSV000084119	26	Q-Exactive	70000	10	<1s	1E5	1E3	5 ppm	0.1	local minimum search (1%, 0.05min, 1%, 1E5, 1, 0.1 - 3.00min)	0.01	0.2	10 ppm, 0.2 min, 75:25	no	2	2	MS2 filter	yes
MSV000084024	251	QToF/ Maxis Impact		8	3Hz MS1/ 10Hz MS2	1E4	1E2	25 ppm	0.01	Baseline cut-off (Min peak height 1.0E4; peak duration range 0.01-1.0 min; Baseline level 1.0E2)	0.01	0.3	25 ppm, 0.2 min, 75:25	no	2	2	only with MS2 or annotation	no
MSV000082045	2249	QToF/ Maxis Impact	17000	10	<1s	1E3	1E2	20 ppm	0.05	Local minimum search (5.0%, 0.05 min, 5.0%, 3000, 2, 0.05 - 2.00 min)	0.01	0.05	20 ppm, 0.1 min, 50:34	yes	2	2	MS2 filter	yes
MSV000084101	411	QToF/ Maxis Impact				2E3	9E1	0.001 m/z or 20 ppm	0.03	Local minimum search (96%, 0.03 min, 5.0%, 2000, 1, 0.0 - 2.00 min)	0.02	0.15	0.0015 m/z or 15ppm, 0.2 min, 2:1	no	2	2	no	no
MSV000083729	298	Q-Exactive	17000		<1s	1E5	1E3	0.005 m/z or 10 ppm	0.02	Local minimum search (1%, 0.05min, 1%, 3000, 1.5, 0.05 - 2.00min)	0.01	0.1	0.01 m/z or 10ppm, 0.2 min, 75:25	no	2	2	no	no
MSV000083772	80	Q-Exactive	17000		<1s	1E5	1E3	0.005 m/z or 10 ppm	0.02	Local minimum search (1%, 0.05min, 1%, 3000, 1.5, 0.05 - 2.00min)	0.01	0.1	0.01 m/z or 10ppm, 0.2 min, 75:25	no	2	2	no	no

Supplementary Table 4: continued

ID	Samples	MS Instrument	MS Resolution	Gradient [min]	Duty Cycle Time	MS^1 threshold	MS^2 Threshold	MS Tolerance	RT Tolerance [min]	Chromatogram Deconvolution Algorithm	MS^2 Paring *m/z* Tolerance	MS^2 Paring RT Tolerance [min]	Feature Aligment (m/z Tolerance, RT Tolerance, m/z : RT Weights)	Duplicate Row Filter	Min. Peaks in a Row	Min. Isotope Pattern Peaks	MS^2 Filtered	Gap Filled
MSV000083601	76	Q-ToF/micrOTOF-QII	18000	25		1E3	1E2	20 ppm	0.02	Local minimum search (1.0%, 0.15 min, 1.0%, 1000, 1.5, 0.10 - 2.00 min)	0.05	0.2	20 ppm, 0.3 min, 75:25	no	2		NA	no
MSV000084045	11	Q-Exactive	17000	5	<1s	1E5	1E3	10 ppm	0.02	Baseline cut-off (Min peak height: 1E5, Peak dur 0.01 - 2 min, Baseline level 1E5)	0.01	0.1	0.01 m/z or 10 ppm, 0.1 min, 75:25	no	2	2	only with MS2 or annotation	yes
MSV000084112	116	Q-Exactive	32000	12.5		1E5	5E2	0.01 m/z or 20 ppm	0.05	Local minimum search (0.01%, 0.04 min, 0.01%, 1E5, 2 0.05 - 0.50 min)	0.01	0.1	0.01 m/z or 20 ppm, 0.3 min, 75:25	No	2		no	yes
MSV000082630	449	Q-Exactive	32000	12.5		2E5	1E2	0.005 m/z or 10 ppm	0.05	Baseline cut-off (Min peak height 6E5, Peak dur 0.05-0.4 min, Baseline level 2E5)	0.01	0.1	0.005 m/z or 10 ppm, 0.3 min, 75:25	no	2	0	no	yes
MSV000084007	45	QToF/5600 Triple TOF		8	<2s	5E2	1E1	20 ppm	0.15	Baseline cut-off (Min peak height 1.8E3, Peak dur 0-3 min, Baseline level 1E3)	0.02	0.15	20 ppm, 0.15 min, 75:25	no	2	2	no	no
MSV000084056	9	Q-ToF/micrOTOF-QII		62		3E3	5E1	20 ppm	0.1	Local minimum search (1.0%, 0.1 min, 1.0%, 4000, 1.2, 0.1 - 2.00 min)	0.02	0.1	20 ppm, 0.1 min, 75:25	no	3	2		no
MSV000084107	129	Q-Exactive plus	35000	18		5E5	5E2	10 ppm	0.1	Local minimum search (30%, 0.1 min, 5.0%, 1500, 2, 0 - 2.00 min)	0.02	0.1	7 ppm, 0.2 min, 2:1	yes	2	2	no	yes
MSV000084063	9	Q-ToF/micrOTOF-QII	18000	25		1E3	1E2	20 ppm	0.1	Local minimum search (1.0%, 0.1 min, 1.0%, 1000, 1.5, 0.1 - 2.00 min)	0.05	0.2	20 ppm, 0.5 min, 75:25	no	2			no
MSV000081832	49	QToF/ Maxis Impact	17000			1E3	1E2	20 ppm	0.05	Baseline cut-off (Min peak height 1.0E3, Peak dur 0.01-3 min, Baseline level 1.0E3)	0.01	0.1	20 ppm, 0.1 min, 75:25	no	2	2	only with MS2 or annotation	yes
MSV000084134	15	LTQ-Orbitrap XL	30000	30		3E4	1E4	10 ppm	0.1	Local minimum search (65%, 0.08 min, 0%, 6E4, 1.5, 0-3 min)	0.2	0.2	0.002 m/z or 7 ppm, 0.2 min, 3:1	no	2	0	only with MS2 or annotation	yes

Supplementary Table 4: continued

ID	Samples	MS Instrument	MS Resolution	Gradient [min]	Duty Cycle Time	MS^1 threshold	MS^2 Threshold	MS Tolerance	RT Tolerance [min]	Chromatogram Deconvolution Algorithm	MS^2 Paring *m/z* Tolerance	MS^2 Paring RT Tolerance [min]	Feature Aligment (m/z Tolerance, RT Tolerance, m/z : RT Weights)	Duplicate Row Filter	Min. Peaks in a Row	Min. Isotope Pattern Peaks	MS^2 Filtered	Gap Filled
MSV000084148	14	LTQ-Orbitrap XL	30000	30		1E4	2E4	10 ppm	0.1	Local minimum search (85%, 0.1 min, 0%, 2E4, 1.4, 0-2 min)	0.2	0.08	0.0015 m/z or 8 ppm, 0.1 min, 3:1	yes	3	0	only with MS2 or annotation	yes
MSV000084158	13	LTQ-Orbitrap Elite	120000	20	<2s	1E4	1E2	10 ppm	0.15	Local minimum search (5%, 0.2 min, 5%, 2E4, 1, 0.02-5 min)	0.01	0.15	10 ppm, 0.2 min, 75:25	no	2	0	MS2 filter	yes
MSV000084170	88	QToF/ Maxis Impact	17000	8		8E2	8E1	20 ppm	0.1	Local minimum search (98.5%, 0.08 min, 0%, 6E3, 1, 0.03-1.5 min)	0.03	0.1	0.01 m/z or 20 ppm, 0.1 min, 75:25	yes	1	2	no	yes
MSV000084118	9	Q-Exactive	17000	10	<1sec	1E5	1E3	10 ppm	0.15	Local minimum search (5%, 0.2 min, 5%, 2E4, 1, 0.02-5 min)	0.01	0.15	10 ppm, 0.2 min, 75:25	no	2	0	MS2 filter	yes

Supplementary Table 5: Description of all statistical measures that were extracted for each dataset.

Measure	Description
Metadata	
MassIVE ID	ID to access the publicly available dataset in the MassIVE repository
Sample origin	Study and sample descriptor (e.g., Standards, Urine,..)
MS type	Mass analyzer type (FTMS or qTOF)
Machine type	Name of the mass spectrometer
Summary of IIMN+FBMN statistics	
Total nodes	All feature nodes (might include nodes without MS^2)
Total nodes with MS^2	All feature nodes with MS^2 spectrum
Library matches (MS^2)	Matches to the GNPS spectral libraries (= annotations)
Ion identities (MS^1)	Nodes with ion identity annotation based on MS^1
Ion identities with MS^2	Nodes with ion identity annotation that have an MS^2 spectrum
MS^2 with ion identity %	Nodes with ion identity relative to total nodes with MS^2
Library matches + ion identity	Library matches with ion identities
Library matches + ion identity %	Relative to all library matches
Ion identity networks	Number of ion identity networks, representing connected ion identities which point to the same neutral molecular mass
All measures below were calculated in regards to nodes with a minimum number of MS^2 signals, filtered with n=0,1,4,6 signals. FBMN and IIMN comparisons are based on results for n=0 (no filter).	
MS^2 scans	Nodes with a spectrum with at least n signals
Annotated	Annotated nodes with at least n MS^2 signals
Annotated %	Relative to all nodes with MS^2 scans with at least n signals
Singletons FBMN / IIMN	Nodes that have no connecting FBMN edge / nodes that have no connecting IIMN edge
Singletons FBMN / IIMN%	Relative to all nodes with MS^2 scans with at least n signals
Nodes reduced by IIN	Redundant nodes that describe the same neutral molecule are reduced by collapsing all ion identities of an IIN into a singular neutral molecule node “M”. An IIN with 3 ions (nodes) would then result in a reduction of 2 nodes for the remaining “M”-node.
Nodes reduced by IIN %	Relative to all nodes with MS^2 scans with at least n signals

Supplementary Table 5: continued

Measure	Description
Remaining nodes after reduction	MS^2 scans - nodes reduced by IIN
Possible new library spectra by IIN	Counts all nodes (MS^2 spectra) without a direct match to the GNPS spectral libraries but with an IIN edge to at least one identified node. The count will be 2 if one or more nodes in an ion identity network are identified by a spectral library match and connected to 2 additional unidentified ion identities with at least n MS^2 signals.
Identification density as the distance to an annotated node (each node only counts once even if connected to multiple annotated compounds) in regards to nodes with a minimum number of MS^2 signals, filtered with n=0,1,4,6 signals. FBMN and IIMN comparisons are based on results for n=0 (no filter).	
Distance 0,1,2,3,4,5 edges	Number of nodes with a minimum distance (0-5 edges) to an identified node with a spectral library match. Comparison between FBMN MS^2-based edges only and IIMN (MS^2+MS^1-based) networks.
Distance 0,1,2,3,4,5 edges %	Relative to all nodes with MS^2 scans with at least n signals (identification density over n edges)
Ion identity networks statistics	
Adduct distribution	List of all ion identities and their occurrence in the dataset
NetID	The network identifier number. All nodes with an ion identity point to a specific netID.
Net size	Number of feature nodes in a network
Nodes with MS^2	Number of nodes with an MS^2 spectrum
Identified	Nodes with a match to the GNPS spectral libraries
All ions	All ion identities (e.g., $[M+H]^+$)
Ions matched library	All ion identities with a library match
Reduction by	Reduction of nodes by collapsing IINs into a single neutral molecule-node. (Nodes with MS^2-1)
Possible new library entries with n signals (n=3,4,6)	If IIN has at least one identified node: List of ion identities with no match to the GNPS spectral libraries but at least n MS^2 signals.

Supplementary Table 6: Links to the GNPS IIMN job pages for each dataset provide options for downloading the results and analyzing them in the GNPS web interfaces. The IIMN workflow parameters are available by job cloning, which further enables reanalysis.

#	MassIVE ID	GNPS Job
1	MSV000082081	https://gnps.ucsd.edu/ProteoSAFe/status.jsp?task=97cc00c7187040db9efeb9558694bed6
2	MSV000084116	https://gnps.ucsd.edu/ProteoSAFe/status.jsp?task=7a896f99dd474cac95e651f83f873ea0
3	MSV000084008	https://gnps.ucsd.edu/ProteoSAFe/status.jsp?task=0a0b1e6d245e43a5a2fc5b3caaf37f3b
4	MSV000084099	https://gnps.ucsd.edu/ProteoSAFe/status.jsp?task=aa077cf9013249de91edd87c27783173
5	MSV000084119	https://gnps.ucsd.edu/ProteoSAFe/status.jsp?task=b56ba768f495427299e62f4aa4efab3e
6	MSV000084024	https://gnps.ucsd.edu/ProteoSAFe/status.jsp?task=c5b6d1782a7842aea1a852c43ab06033
7	MSV000082045	https://gnps.ucsd.edu/ProteoSAFe/status.jsp?task=e8c8622636854be3a3e32fb08bfc3536
8	MSV000084101	https://gnps.ucsd.edu/ProteoSAFe/status.jsp?task=d97eb96c693d43c08fd0c9999cc2ada7
9	MSV000083729	https://gnps.ucsd.edu/ProteoSAFe/status.jsp?task=328bbf4d8a5042b89de7c3042c668cab
10	MSV000083772	https://gnps.ucsd.edu/ProteoSAFe/status.jsp?task=db2749854d9f4b369b2ea2cfd6de0b90
11	MSV000083601	https://gnps.ucsd.edu/ProteoSAFe/status.jsp?task=79a0bf62e64e4e5fa284e37a3bb5f9a2
12	MSV000084045	https://gnps.ucsd.edu/ProteoSAFe/status.jsp?task=4a5a472d271f4234991ee9f8012b71ea
13	MSV000084112	https://gnps.ucsd.edu/ProteoSAFe/status.jsp?task=8ac11424b0844dd2822b97006c46427f
14	MSV000082630	https://gnps.ucsd.edu/ProteoSAFe/status.jsp?task=b5a98589efb24810897944cfb801d800
15	MSV000084007	https://gnps.ucsd.edu/ProteoSAFe/status.jsp?task=2ee81a5ed11441af822e211c775d6a3d
16	MSV000084056	https://gnps.ucsd.edu/ProteoSAFe/status.jsp?task=c0cdcb675607454c8c4c2985e03bdc01
17	MSV000084107	https://gnps.ucsd.edu/ProteoSAFe/status.jsp?task=7ba1a6a1a0934596bac5dfaf55290f81
18	MSV000084063	https://gnps.ucsd.edu/ProteoSAFe/status.jsp?task=2b93b1072d84497a86d92364e8c515ce
19	MSV000081832	https://gnps.ucsd.edu/ProteoSAFe/status.jsp?task=9eacca5cd179499ea35ba15c60b14c0d
20	MSV000084134	https://gnps.ucsd.edu/ProteoSAFe/status.jsp?task=0308d797c31a477292fe009030d03da7
21	MSV000084148	https://gnps.ucsd.edu/ProteoSAFe/status.jsp?task=a5480529261b4a13bb867f2edad1dcbe
22	MSV000084158	https://gnps.ucsd.edu/ProteoSAFe/status.jsp?task=de2afe9697aa450fba5b6c0b0d876bbd
23	MSV000084170	https://gnps.ucsd.edu/ProteoSAFe/status.jsp?task=3f5c4666eb9f4f42aedf2e36bead34a5
24	MSV000084118	https://gnps.ucsd.edu/ProteoSAFe/status.jsp?task=fb1461d9099042529511e4c3ccb32572

Supplementary References

1. Aron, A. *et al.* Native Electrospray-based Metabolomics Enables the Detection of Metal-binding Compounds. doi:10.1101/824888.
2. Jagels, A. *et al.* Exploring Secondary Metabolite Profiles of Stachybotrys spp. by LC-MS/MS. *Toxins* **11**, (2019).
3. Horai, H. *et al.* MassBank: a public repository for sharing mass spectral data for life sciences. *J. Mass Spectrom.* **45**, 703–714 (2010).
4. Wang, M. *et al.* Sharing and community curation of mass spectrometry data with Global Natural Products Social Molecular Networking. *Nat. Biotechnol.* **34**, 828–837 (2016).
5. Vargas, F. *et al.* Protocol for Community-created Public MS/MS Reference Library Within the GNPS Infrastructure. *bioRxiv* 804401 (2019) doi:10.1101/804401.
