# Supplementary material for: Ion identity molecular networking for mass spectrometry-based metabolomics in the GNPS environment
Source: Nat Commun. 2021 Jun 22;12:3832. doi: 10.1038/s41467-021-23953-9 (PMC8219731; doi:10.1038/s41467-021-23953-9)
Supplement: Supplementary file 3 — Description of Additional Supplementary Files [file 41467_2021_23953_MOESM3_ESM.docx]

**Description of Additional Supplementary Files**

**File Name:** Supplementary Data 1

**Description:** Comprehensive statistical results of IIMN on 24 experimental datasets.
