# Supplementary material for: Ion identity molecular networking for mass spectrometry-based metabolomics in the GNPS environment
Source: Nat Commun. 2021 Jun 22;12:3832. doi: 10.1038/s41467-021-23953-9 (PMC8219731; doi:10.1038/s41467-021-23953-9)
Supplement: Supplementary file 5 — Reporting Summary [file 41467_2021_23953_MOESM5_ESM.pdf]

Corresponding author(s): Pieter C Dorrestein
Last updated by author(s): Apr 21, 2021

Reporting Summary

Nature Research wishes to improve the reproducibility of the work that we publish. This form provides structure for consistency and transparency in reporting. For further information on Nature Research policies, see our Editorial Policies and the Editorial Policy Checklist.

Statistics

For all statistical analyses, confirm that the following items are present in the figure legend, table legend, main text, or Methods section.

n/a	Confirmed	
☐	☒	The exact sample size (*n*) for each experimental group/condition, given as a discrete number and unit of measurement
☐	☒	A statement on whether measurements were taken from distinct samples or whether the same sample was measured repeatedly
☐	☒	The statistical test(s) used AND whether they are one- or two-sided<br>*Only common tests should be described solely by name; describe more complex techniques in the Methods section.*
☐	☒	A description of all covariates tested
☐	☒	A description of any assumptions or corrections, such as tests of normality and adjustment for multiple comparisons
☐	☒	A full description of the statistical parameters including central tendency (e.g. means) or other basic estimates (e.g. regression coefficient) AND variation (e.g. standard deviation) or associated estimates of uncertainty (e.g. confidence intervals)
☐	☒	For null hypothesis testing, the test statistic (e.g. *F*, *t*, *r*) with confidence intervals, effect sizes, degrees of freedom and *P* value noted<br>*Give P values as exact values whenever suitable.*
☐	☒	For Bayesian analysis, information on the choice of priors and Markov chain Monte Carlo settings
☐	☒	For hierarchical and complex designs, identification of the appropriate level for tests and full reporting of outcomes
☐	☒	Estimates of effect sizes (e.g. Cohen's *d*, Pearson's *r*), indicating how they were calculated

Our web collection on statistics for biologists contains articles on many of the points above.

Software and code

Policy information about availability of computer code

Data collection	The IIMN workflow is available as an interface on the GNPS web platform (https://gnps-quickstart.ucsd.edu/featurebasednetworking). The workflow code is open source and available on GitHub (https://github.com/CCMS-UCSD/GNPS_Workflows). It is released under the license of The Regents of the University of California and free for non-profit research (https://github.com/CCMS-UCSD/GNPS_Workflows/blob/master/LICENSE). The workflow was written in Python (ver. 3.7) and deployed with the ProteoSAFE workflow manager employed by GNPS (http://proteomics.ucsd.edu/Software/ProteoSAFe/).<br>The source code of all modules which were implemented into MZmine, e.g., the Export for IIMN module, the metaCorrelate grouping module, the ion identity networking modules, and the results and spectral library generation module, is available at http://mzmine.github.io/iin_fbmn under the GNU General Public License.<br>The source code for the custom GNPS export functions for XCMS is available at https://github.com/jorainer/xcms-gnps-tools under the GNU General Public License. The workflow was tested with CAMERA version 1.46.0,<br>The source code of MS-DIAL (IIMN support starting at version 4.1) is availalbe at http://prime.psc.riken.jp/compms/msdial/main.html
Data analysis	Cytoscape v3.7.1, MZmine (build 2.37.1_IIN_17.7_LS), GNPS FBMN version 18, Microsoft Excel 365 (version 2103)

For manuscripts utilizing custom algorithms or software that are central to the research but not yet described in published literature, software must be made available to editors and reviewers. We strongly encourage code deposition in a community repository (e.g. GitHub). See the Nature Research guidelines for submitting code & software for further information.

Data

Policy information about availability of data

All manuscripts must include a data availability statement. This statement should provide the following information, where applicable:

- Accession codes, unique identifiers, or web links for publicly available datasets
- A list of figures that have associated raw data
- A description of any restrictions on data availability

All raw (.raw) and centroided peak picked (.mzXML or .mzML) mass spectrometry data as well as processed data (.mgf and .csv) and ion identity molecular networks are available through the MassIVE repository (massive.ucsd.edu). Individual MassIVE dataset identifiers are listed in Supplementary Table 1. Dataset metadata and MZmine processing parameters are available in Supplementary Table 45. Links to IIMN job pages for each dataset are listed in Supplementary Table 6 with options for downloading or online analysis of results. Job cloning provides access to all parameter values and to reproducible data reanalysis. The statistical results on all 24 datasets are available in Supplementary File SI_IIMN_dataset_statistics.xlsx. The ion identity statistics on different MS2 spectral databases are available as Supplementary File SI_IIMN_spectral_library_anaylsis.xlsx. The IIMN-based MS2 spectral libraries of propagated spectral entries can be used within GNPS or downloaded for free. Source data are provided with this paper.
The IIMN-based MS2 spectral library from experimental datasets:
https://gnps.ucsd.edu/ProteoSAFe/gnpslibrary.jsp?library=GNPS-IIMN-PROPOGATED
The IIMN-based MS2 spectral libraries from 2 datasets generated for the NIH Natural Products Library (NIH NPAC ACONN) were merged into the existing manually created GNPS library (NIH Natural Products Library Round 2), available on GNPS:
https://gnps.ucsd.edu/ProteoSAFe/gnpslibrary.jsp?library=GNPS-NIH-NATURALPRODUCTSLIBRARY_ROUND2_POSITIVE
Individual download links for the two libraries are:
Q-Exactive dataset: https://gnps.ucsd.edu/ProteoSAFe/status.jsp?task=c39d788d30f9408a9e53e68ec84868c6
Q-TOF-MS dataset: https://gnps.ucsd.edu/ProteoSAFe/status.jsp?task=904e6d42b5024c5cacef6dd86f02b714

The datasets are available with their accession IDs in the MassIVE repository:
MSV000082081 (https://massive.ucsd.edu/ProteoSAFe/dataset.jsp?task=f65bfac6208a436fab483cd284f52a33), MSV000084116 (https://massive.ucsd.edu/ProteoSAFe/dataset.jsp?task=81538c459d0447ef972d267d2fb0111d), MSV000084008 (https://massive.ucsd.edu/ProteoSAFe/dataset.jsp?task=93f45e7eba2e456083a35a92610fff52), MSV000084099 (https://massive.ucsd.edu/ProteoSAFe/dataset.jsp?task=6ab3caf2593e4310a6516357f0657aeb), MSV000084119 (https://massive.ucsd.edu/ProteoSAFe/dataset.jsp?task=fb0ca514a168427d817a29ba90c4b9f2), MSV000084024 (https://massive.ucsd.edu/ProteoSAFe/dataset.jsp?task=ce840c3053d04c9b8ef1d6daf7068a98), MSV000082045 (https://massive.ucsd.edu/ProteoSAFe/dataset.jsp?task=64e3aacbbbfd4b8681e7e788cb6b16fa), MSV000084101 (https://massive.ucsd.edu/ProteoSAFe/dataset.jsp?task=3b3d495b93c047adb2d8d25bf6205dc9), MSV000083729 (https://massive.ucsd.edu/ProteoSAFe/dataset.jsp?task=552dca562b1e4dd8a2ddbeb5e162d290), MSV000083772 (https://massive.ucsd.edu/ProteoSAFe/dataset.jsp?task=eaf6dfcfddc94612a4aa85e9c2e308db), MSV000083601 (https://massive.ucsd.edu/ProteoSAFe/dataset.jsp?task=48dc7250e98e45ffb988cfd353d53a3d), MSV000084045 (https://massive.ucsd.edu/ProteoSAFe/dataset.jsp?task=43e2ce9cd85c47678bf3250dbb9e047b), MSV000084112 (https://massive.ucsd.edu/ProteoSAFe/dataset.jsp?task=a03639b5a08d42d283b714b64146087c), MSV000082630 (https://massive.ucsd.edu/ProteoSAFe/dataset.jsp?task=90cefc55f6464e20a873e471c5b962e1), MSV000084007 (https://massive.ucsd.edu/ProteoSAFe/dataset.jsp?task=c809f27dd91445f68c8cc522936119f4), MSV000084056 (https://massive.ucsd.edu/ProteoSAFe/dataset.jsp?task=bea6f8e8a5f14074bd884ee6dd659ab9), MSV000084107 (https://massive.ucsd.edu/ProteoSAFe/dataset.jsp?task=469779645cd94f159280a88e07c9cf7a), MSV000084063 (https://massive.ucsd.edu/ProteoSAFe/dataset.jsp?task=9d478e5f428443ff829f3decbd5759d6), MSV000081832 (https://massive.ucsd.edu/ProteoSAFe/dataset.jsp?task=3720644eead2496f8b48f6c09b8d4790), MSV000084134 (https://massive.ucsd.edu/ProteoSAFe/dataset.jsp?task=1ce9d2290ad04fc4bb4acf143ffe0b92), MSV000084148 (https://massive.ucsd.edu/ProteoSAFe/dataset.jsp?task=af4486f029a546d09e1dd572b66ddac5), MSV000084158 (https://massive.ucsd.edu/ProteoSAFe/dataset.jsp?task=1fedc205b6104024a901297a9c0ef151), MSV000084170 (https://massive.ucsd.edu/ProteoSAFe/dataset.jsp?task=17709af0ba294e1387192091a1c541e7), MSV000084118 (https://massive.ucsd.edu/ProteoSAFe/dataset.jsp?task=a13ac7b7be10421c8a168176752cc586)

Field-specific reporting

Please select the one below that is the best fit for your research. If you are not sure, read the appropriate sections before making your selection.

☒ Life sciences ☐ Behavioural & social sciences ☐ Ecological, evolutionary & environmental sciences

For a reference copy of the document with all sections, see nature.com/documents/nr-reporting-summary-flat.pdf

Life sciences study design

All studies must disclose on these points even when the disclosure is negative.

Sample size

This study includes 24 public datasets with different sample sizes ranging from 7 to 2249 samples/LC-MS2 runs. The datasets were chosen to cover a broad range of instrument and sample types. Detailed information including accession numbers for downloading of the data and metadata is provided in Supplementary Table 6.

Data exclusions

No files or studies have been excluded.

Replication

The general concept of IIMN was validated by triplicate injection of a mixture of standards with different post LC-column infusion of salt solutions. Na-acetate and NH4-acetate were chosen to induce the formation of Na and NH4 adducts, which was directly reflected by significant changes (Welch two-samples t-test, $p<0.001$) in the relative abundance of ion identities across the sample groups. Triplicate injections were chosen to verify the reproducibility of the LC-MS separation and ionization and the feature finding procedure that extracts feature areas.

Randomization

The triplicate injections with post column salt infusion were measured for a specific condition (solution and concentration) and then purged

Randomization	with the new solution before injection. In this experimental setup we needed controlled conditions to induce adduct formation with a clear ionization trend for [M+Na]+ and [M+NH4]+ to verify the ion identities.
Blinding	Datasets were assigned a numerical identifier which was linked to study and sample metadata.<br>The salt infusion experiments were not blinded as they aim to provide an initial validation of ion identities in IIMN by comparing the abundance of different ion identities across different post column salt infusion conditions. Here, the infusion of different cations, namely Na+ and NH4+, was expected to boost the corresponding ion adducts.

Reporting for specific materials, systems and methods

We require information from authors about some types of materials, experimental systems and methods used in many studies. Here, indicate whether each material, system or method listed is relevant to your study. If you are not sure if a list item applies to your research, read the appropriate section before selecting a response.

Materials & experimental systems

n/a	Involved in the study
☒	☐ Antibodies
☒	☐ Eukaryotic cell lines
☒	☐ Palaeontology and archaeology
☒	☐ Animals and other organisms
☒	☐ Human research participants
☒	☐ Clinical data
☒	☐ Dual use research of concern

Methods

n/a	Involved in the study
☒	☐ ChIP-seq
☒	☐ Flow cytometry
☒	☐ MRI-based neuroimaging
